# AGE-RELATED HEARING LOSS IN COMMUNITY PHARMACY: BARRIERS AND FACILITATORS TO COMMUNICATION

**DOI:** 10.1093/geroni/igad104.1472

**Published:** 2023-12-21

**Authors:** Penny Lewis, Shanice Thomas, Jane Griffiths, Denham Phipps, Gabrielle Saunders, Christopher Todd

**Affiliations:** The University of Manchester, Manchester, England, United Kingdom; University of Manchester, Manchester, England, United Kingdom; University of Manchester, Manchester, England, United Kingdom; University of Manchester, Manchester, England, United Kingdom; University of Manchester, Manchester, England, United Kingdom; University of Manchester, Manchester, England, United Kingdom

## Abstract

Pharmacists have an important role in primary care, communicating with people and ensuring safe and appropriate medication use. However, inadequate communication is a barrier to the delivery of effective care for people with hearing loss. This study sought to explore factors that facilitate and impede communication with people with age-related hearing loss (presbycusis) in the community pharmacy, in order to identify solutions to improve these interactions. Online semi-structured interviews with people with age-related hearing loss (presbycusis), older people (>50 years old) without hearing loss, and online focus groups and interviews with community pharmacists were conducted. Data were analysed using the framework method. Sixteen people with age-related hearing loss, three older people without hearing loss and eight community pharmacists took part. Participants described a multitude of environmental barriers to communication and person-centred pharmaceutical care such as heavy workload, lack of privacy, noise levels and Covid-19 safety measures. There was a perception among participants that their hearing loss is not relevant to the community pharmacy setting and that more could be done to signify that a pharmacy recognises the needs of those with hearing loss, furthermore, participants discussed their limited interaction with pharmacy personnel. There were varying perceptions about communication and levels of awareness among pharmacists about the key facilitators to communication. Greater interdisciplinary collaboration to develop and implement strategies/adaptations tailored to the needs of people with hearing loss would support the engenderment of hearing-friendly community pharmacies and the safe use of medicines.

